# Mental health problems in Somalia after decades of humanitarian crises: a qualitative exploration of perceptions and experiences

**DOI:** 10.1186/s41182-024-00618-z

**Published:** 2024-09-09

**Authors:** Md Manirul Islam, Abdiwali Ahmed Siyad, Sk Md Mamunur Rahman Malik

**Affiliations:** 1 World Health Organization Country Office, Mogadishu, Somalia; 2 World Health Organization Eastern Mediterranean Regional Office, Cairo, Egypt

## Abstract

**Background:**

Humanitarian crises increase the risk of mental health problems. Somalia has been affected by conflict, insecurity, and economic turmoil for over three decades, as well as climatic shocks. However, 80–90% of Somalis who have mental health problems do not have access to good-quality, and affordable mental health care. To develop an evidence-based, effective, equitable, and humane programme for mental health, we need to have a holistic understanding of mental health problems and care in relation to people’s perceptions, experiences, and behaviour related to mental health.

**Methods:**

We undertook a qualitative study to explore Somalis’ perceptions and experiences of mental health problems. We conducted three key informant interviews, two in-depth interviews, nine focus group discussions, 12 observations in private and public health facilities and more than 12 informal discussions. We used case vignettes translated into Somali during our discussion. We also studied three cases with experience of mental health problems to understand care-seeking behaviour and the experiences with services available.

**Results:**

Somalia has been moving from a traditional pastoral nomadic lifestyle to a settled one. A strong informal support system exists in the community within clans or family relations. Armed conflict often among clans, natural disasters, and khat use are the three main factors affecting mental health. The prevalence of mental problems is likely greater than is evident. It is perceived that about 95% of people suffering from mental illness remain outside of appropriate care. Few people seek care for mental health problems because they are not aware of it and because it is highly stigmatized and neglected. Those who do seek care usually go to traditional healers because of culture and cost. Resources for mental health care are grossly inadequate with a limited and often poorly trained workforce. At least two levels of barrier to mental health care exist, at the individual/family level (e.g. poor awareness of mental health and stigma) and service provider level (e.g. lack of staff and limited ability to diagnose, treat, or refer persons with mental health problems and stigma). No tool or evidence-based programme is available to address these barriers.

**Conclusion:**

A qualitative data-driven mental health programme that addresses all these issues is needed with more trained mental health professionals. Given the stigma about mental health problems, there is also a need for a tool to raise awareness about mental health and the importance of mental health care among both the public and health workers.

## Background

Good mental health enables us to connect and work well, deal with stress, and prosper. It is a ‘complex continuum’, ranging from feeling the best to unbearable states of loss of insight, great misery, and ‘emotional pain’ [1]. Armed conflicts, natural disasters and humanitarian crises have been shown to increase the burden of mental health problems more than twofold compared with the general population, which stands at 10%. Most of the mental health problems (75%) affect low- and middle-income countries [2–4]. Somalia has one of the world’s most enduring humanitarian crises. Decades of conflict, insecurity, loss of livelihood, economic turmoil and poor living conditions have eroded the resilience of the people which has led to increased psychosocial distress. At the same time, prolonged crises have resulted in a weakened, fragmented, and under-funded health system which struggles to serve the health needs of the people [4–7].

Although nationally representative data on the burden of mental illness are not available for Somalia, a recent epidemiological study in three conflict affected zones revealed a high prevalence of mental health problems (76.9%) among the study participants, most of whom (68.1%) were younger than 35 years [7]. The study also identified a prevalence of substance use disorder of 50.6% and all participants reported a poor quality of life [8]. Despite these figures, 80–90% of Somalis who have a mental health problem do not have access to good-quality, affordable mental health care. Most people (85%) sought help from a Koranic or a traditional healer at least once. The average time for people with mental health problems to make their first visit to a health facility to seek care is 3.5 years [9]. Furthermore, people with mental health problems experience widespread human rights violations, discrimination, stigma, and consequent social isolation [10, 11].

Given the reported high burden of mental health problems in the country, the time lag for seeking care, and the lack of data, we aimed to explore Somalis’ perceptions and experiences of mental health problems. This information may help the country to build a sustainable, culturally appropriate programme for mental health care that can be delivered in an integrated manner along with other health services at the primary health care (PHC) level.

As any programme for mental health care needs to be ‘evidence-based’, ‘effective, efficient, equitable and humane’, we need to understand what’s going on in people’s minds and the perspectives they are in [12]. A qualitative approach would allow us to understand people’s perceptions, experiences, and behaviours related to mental health and problems in their natural settings. This is particularly true for Somalia because it is an extremely security-compromised setting and unlike the quantitative approach, the qualitative paradigm can be flexible, valid, and can be adjusted to the prevailing situation [13]. Moreover, a similar study design was used in another African country for a largely similar study objective [14] and as such we considered our qualitative approach to be an appropriate one since it will take into account different stakeholders’ views and cultural contexts [15] at the same time. To our knowledge, there is no such qualitative study carried out in Somalia except in Somaliland to understand the situation and develop appropriate mental health programmes.

## Methodology

We followed a qualitative design and our approach largely matched the grounded theory of qualitative paradigm as we explored to understand various issues relevant to mental health problems and its care and why and how underneath [16].

We carried out the study mainly in Puntland and collected data from mid-February until mid-September 2023. We also collected data from other areas of Somalia such as Hudur and Deyniile in southern Somalia, and Daryeel, Hidho and Hargeisa in Somaliland in the north-west of the country to understand the situation from valid and broader perspectives. We chose Puntland as our primary study site because it was comparatively safer, and access was easier compared with other areas. We used a convenience sampling strategy to select our respondents for security reasons [17].

We structured three different interview guides to collect data from health personnel, traditional or Qur’anic healers and other stakeholders (Table 1). We asked subsequent supplementary questions based on the responses of the interviewees to the initial questions.

**Table 1:** Interview guides (for formal interviews and discussions) and case vignette

A. Interview guide (Health Personnel) -What are the common mental disorders -What are common symptoms of patients with Anxiety, Depression, schizophrenia, bipolar disorder -What are the types of treatment and psychotropic medicines available -What is the cost of psychotherapy per hour (affordable for ordinary Puntlander?) -What proportion of mental disorder patients get treatment and their sociodemographic characteristics -Why people come to you for treatment - How many of them are referred -What are the challenges -Those who are not taking treatment what would be the cause -What are the perceptions about the prevalence and type of mental disorder?	
B. Interview guide (Traditional or Quranic healers) -When you think of mental disorders what would come to your mind; give an example. -When will you think the person may have mental disorders (Symptoms) A case vignette would be presented**.** -What type of problem s/he is suffering from? -What do you think about the cause? -How does your treatment work? -What type of people come to you (economic, educational and religious convictions)? -Why do they come to you? -What proportion of people get better with your treatment? -What do you do if your treatment does not work? -How about referring the patient to a mental health treatment centre? -Do you have any idea where the treatment is available?	
C. Case vignette of depression as an example Fazil is a 10-year-old boy whose teachers see that he has been unhappy for the past 3 months. He often cries in class and seems very emotional. He also seems confused and unable to focus on even simple tasks. He is not motivated to finish his work or participate in class discussions. He seems to have lost a lot of weight. At home, his parents noticed he was upset and quickly prompted by his sister. He complains of being very tired, unable to focus and lacking the motivation to do anything. .	

We translated all these interview guides into Somali languages. We carried out three key informant interviews (KI1s) with government focal persons for mental health, one at the state level (face to face) one at the federal level (online) and one with a qualified psychiatrist (online) working in the capital, Mogadishu. We conducted two in-depth interviews (IDIs), one each with a PHC supervisor and a founder of a private residential facility. We also held nine focus group discussions (FGDs) with nurses and other health personnel working in outreach and PHC centres, staff of an international organization (support personnel, doctors and public health personnel), psychologists working in private facilities, and doctors and administrators of a private hospital (Table 2). All the FGDs were held in the workplace of health care personnel (e.g. outreach centre, PHC centre) except the one with psychologists which we held at the WHO Puntland office.

**Table 2:** Socio-demographic characteristics of the FGD respondents (n=41)

Variable	No.	% (*n*=41)
Age, in years		
18–25	9	22
26–35	17	41
36–60	13	32
61–73	2	5
Sex		
Female	17	41
Male	24	59
Total years of education		
5–10	3	7
11–16	20	49
17–20	16	39
21–22	2	5
Total number of family members		
5–6	7	17
7–10	18	44
11–20	14	34
21–27	2	5
Monthly household running costs, in US$		
200–1000	21	51
1001–1500	11	27
1501–2500	6	15
2501–3000	3	7

The median duration of recorded interviews and discussions was 36.8 min (range 13.5–87.7 min). We had to leave one FGD with internally displaced persons (IDPs) just as we started as security personnel did not allow us to continue. When we expressed our desire to talk to people in the community, the security officer told us that talking to people using personal protective equipment with armed escorts could endanger the people we wanted to talk to.

We also carried out more than 12 informal discussions (IDs) with nurses and community health workers in outreach and PHC centres, doctors in both private and public health facilities, psychiatrists, psychologists, an imam, a traditional healer, a youth leader, relatives of patients with experiences of mental health problems and a senior administrator of the health ministry in different study sites. We conducted three KIIs, four FGDs and eight IDs in English. The remaining interviews and discussions were held in Somali language.

We used case vignettes which are case scenarios (translated into Somali) of schizophrenia, depression (Table 1), anxiety, attention deficit hyperactivity disorder (ADHD), and bipolar mood disorder to explore the perception of the health workers about these mental health problems. The original case scenarios were taken from mental health in schools: a manual by WHO [18]. Additionally, we also studied three clinical cases of mental health problems; two men one with schizophrenia, and the other with depression and one woman with possible postpartum psychosis to understand their and their family members' perception of their illness and care-seeking behaviour and experiences with the services available in the existing health care facility first hand and in detail. We used Somali nationals to collect data from the respondents of these case studies (CSs). We used multiple methods and respondents to ensure the credibility of our findings as mentioned by Korstjens and Moser [19]. We also observed four PHC centres, two rehabilitation centres (known as *ilaaj*), three regional hospitals, one outreach centre, one private mental health facility and one private hospital to understand ‘what’s going on’ there. We kept notes of our activities with dates. These informal approaches and unstructured observations complemented our interviews and discussions and helped in data saturation and triangulation.

We transcribed the data using recording software and entered data, including the field notes, as documents in ATLAS.ti, 23.1.2.0 for Windows and coded the data [20]. The software transcription was not satisfactory as it was sensitive to the accents of the respondents and translators. For example, the word psychiatrist was transcribed as ‘likeartist’, ‘sickest’, ‘catalyst’, ‘cigarettes’, ‘sympatric’ and neurosurgeon as ‘your surgeon’ and psychotherapy as ‘secondary beauty’ because of the different accents of the respondents. We therefore listened to all 14 audio or video recordings at the same time as reviewing the field notes. We took relevant segments of the recordings, compared them with our field notes and what we had already found, and categorized them under different themes. We then reviewed the text to check its consistency and accuracy. Finally, we refined the concepts and developed an explanatory theory to understand the situation related to mental health problems and their treatment. We mostly followed the editing style in the analysis proposed by Miller and Crabtree as we did in another study [21]. We compared the data we had analysed before with the data later and gave our interpretative insights. This fits with the inductive way of analysis. We edited some of the quotes by changing one or two words to make sense and facilitate understanding. We also used an identifier and time locator at the end of the quote to mark where the quote was taken from.

## Results

We describe the key findings under two main sections, context and mental health and the themes within each.

### Context

Exploring the context is important to understand the perception and behaviour relevant to mental health problems of people and different stakeholders. This understanding could help to design an appropriate programme for mental health problems [12].

### Historical context

The informal discussions revealed that Somalia had undergone a transition from a traditional Somali pastoral and nomadic life to a settler life. At one time, 75% of the Somali population were nomadic travelling for months and sleeping as soon as it became dark. Then, many started settling. The ongoing urbanization and the need of settlers for meat and milk and the nomads for essential commodities brought the two groups closer. The nomads started to graze their camels closer to settler areas and often established villages nearby. The settler life was easier than the nomadic life and gradually more nomads opted for it.

### Men’s position

In Somalia, culturally, men are viewed as strong and protectors. Many respondents thought that the use of mindfulness does not work in Somalia as people might consider it a weak approach.

*“Somali culture is little bit harsh. Because of the normally nomadic culture, it’s very tough. If they see you, you are not (strong), if you look weak, and they call it if you have hard time. So, every one of us try to hide what feeling reality and show quite fix. That’s our culture. It’s very tough culture”.* (FGD-5.1-10:43-11:05min)*.*

### Informal support system and sin tax

A strong informal support system existed in the community either through clans or relations which stemmed from the nomadic background. Many participants agreed with the need for family members and their close relatives as noted by a counsellor.

“*…. People, they have all the structures. We can focus on protective factors. Somali people are very social, so social connection, social network. We can also strengthen social network”*. (FGD-3.1-22:55-23:10min)

The one residential facility for mental health in Puntland was established by a collective effort of a religious leader, clan leader, volunteers and donations from patients who were treated there.

However, a new way of generating funds for mental health care has emerged, a so-called *sin tax*. This is a tax on khat (chewing plant used as a stimulant) of US$ 0.05 for each kilogram imported. The Somaliland administration raised US$ 2 000 000 in 2022 from this tax and donated the entire amount to the Department of Mental Health of the Somaliland Ministry of Health to support care for people with mental health problems.

### Armed conflict, natural disasters and khat use

All but one of the 17 regions in Somalia was facing some type of emergency at the time of the study, either armed conflict, natural disasters, clan issues or a high prevalence of khat chewing. During June–October 2023, 255 explosions from improvised explosive devices were reported claiming 692 lives and five suicide bombings occurred killing more than 100 people [22]. These perpetual security incidents are one of the main contributing factors to the worsening mental health problems in the country. A senior public health official substantiated this.

*“Tribalism, terrorism [Interviewer: Tribalism?] yeah, clan. They undermine each other, they kill each other, they humiliate each other. Somali contexts are tough*. *You name*.” (IDI-1-5:50- 6:10 min)

The situation in relation to emergencies is different across different regions of Somalia. This was reflected in the key informant interview with a psychologist working in mental health in Somalia.

*“Every area is different, what they are facing from which. For now, you are in Garowe. So, people are more stable there. They have food security, so they don’t have that problem, maybe if they have (problem in) education. What increases the level of mental health diseases; conflict, unemployment, lack of education, lack of work and everything. But we cannot generalize. … If you look to Mogadishu, all people are suffering from trauma because we have mass casualties, every now and then there is explosions happen, there is, we are living in a stressful situation. In other areas, they have, maybe drought or other emergencies* there; they are facing different forms. (KII-1-25:44-28:10mins)

However, the situation often changes and sometimes abruptly. For example, during a field visit while in Garowe, a political conflict turned violent and reportedly claimed 27 lives. Additionally, 73 patients were admitted to Garowe General Hospital with injuries because of a gunfight at Las Anod, the administrative capital of Sool, which is a contested area between Somaliland and Puntland. The director of the hospital claimed that 68% (50/73) of the injured were with mental health problems. We also observed the worried faces of women waiting outside the hospital for family members injured by the armed violence.

The law on gun possession is not the same across the regions; a gun license is not necessary in some places. One of the respondents recalled an incident where a small boy hid himself when he heard a gunshot. When asked why he was hiding, the mother said his father had been killed by a gunshot. The child was a typical case of post-traumatic stress disorder (PTSD). This incident shows how armed conflict is taking a toll on the mental health of Somali people, especially on the children and the young people. The situation continues to be further complicated because of environmental and climatic factors such as droughts and excessive rainfall because of El Nino, which are also affecting the mental health of the people.

One of the most important risks for mental health problems in the country is the use of khat. Evidence shows that khat use is associated with mental health illness [23, 24]. The proportion of people chewing khat in Somalia varies across different regions, with the highest use in Somaliland with around 60% of males and 5% of females chewing khat. The government allows the import of khat from Ethiopia and Kenya as it earns large tax revenues. One key informant mentioned a hospital in Mogadishu which has a department for mental health with 100 beds. She said, “… 94% of those beds, we can say are occupied by those with substance abuse problem.”

The nomadic pastoral male-dominating inheritance and clan-based identity while ensured some informal support, it often resulted in armed conflicts among different clans. The situation was worsened by climatic conditions and widespread khat use. Consequently, people were always at risk of suffering from mental health problems.

### Mental health

#### Neglect of mental health

“The concept of mental health is very new” commented a key informant who is a psychiatrist. Mental health is not given adequate attention in the medical education system in Somalia and few medical schools provide opportunities for mental health internships for junior doctors, as explained by a key informant.

*“While most are not, most of them, they don’t do any practicum, they don’t do clerkship or internship. They only do the theoretical part*” (KII-3 39:23-39:38min)

This is also reflected in an interview with a psychologist and focal person for mental health at the federal government level.

*“...a basically neglected area and for the last, we can say, for the last 20-30 years it is a neglected area and because of that there is not much of infrastructure regarding the mental health at country level with lots of the emergencies now going on, a bit increase and the, and the discrimination in society related to mental diseases. Common mental causes in other facilities are psychosis and neurological problems but majority of that facilities, 90% are dealing with mental health problems of people who are dealing with substance use. And there is no awareness in the community about that problem.*” (KII-1-1:48-~3:0 mins)

Another problem related to poor mental health could be the use of steroids by women to lighten their skin colour; weight gain is a side-effect of these drugs. In informal discussions with a female psychiatrist and doctors, they noted that around 30% of women in Somalia use steroids for cosmetic reasons. This may explain why some women are suffering from depression and psychosis as studies have found a positive association between these disorders and the use of steroids [25].

#### Perceived magnitude of mental health problems

No quantitative estimates are available of how many people are suffering from mental health conditions across Somalia. Mental health conditions can be seen as an iceberg; the actual prevalence is likely much higher than recorded. A counsellor in private practice in Somalia mentioned the main mental health problems affecting health-care seekers.

*“Most of the time, people who come to our office are mainly, we are dealing with depression, anxiety, and some, most of the cases as you know, most of population in Somalia, they experience trauma*.” (FGD-3.2- 00:54:01:45min)

Another respondent, a male psychologist, also mentioned symptoms of trauma such as withdrawal, isolation, avoidance, lack of trust and anger, and drug addiction as a coping mechanism.

Somali people, especially teenagers and young people, want to leave Somalia to escape the trauma. Failing to leave Somalia or thinking about how to leave often resulted in mental health problems. These young people are known as *buufi*, that is people to whom nothing else matters except leaving Somalia and the precarious situation there [26], as noted by a nurse manager.

*, “Many people now are complaining to stay in Somalia. They want to go abroad. Going abroad is a part of mental problems”* (IDI-2-11:35-11:41min).

The burden of mental health problems could be higher in Mogadishu than in the north of the country (Puntland and Somaliland) because armed conflicts are more prevalent in the south. A respondent, a doctor in a private hospital, talked about Galkayo, the third largest city and the capital of Mudug region, where armed conflict has been ongoing for the past 10 years, *“If ten patients come to you, 8 or 7 of them complaining mental health problem. But in Garowe, it is stable* (in terms of armed conflict)”. However, during our study, Garowe also faced armed conflict. One of the key informants gave his view on the perceived prevalence of mental health problems.

“*I will, I will say with (substance) use increasing, maybe 40% like that. But I am not sure. [Interviewer: 40% in Mogadishu?] Yeah. But I am not calculating, right? Because, in Somaliland, they don’t have conflict, they have excessive khat chewing.*” (KII-1-28:05-28:28min)

#### Perception of mental health problems

People in general are not aware of mental health problems, nor do they know where to seek help. Our informal discussion revealed that around 95% of those needing care were not receiving proper treatment.

*“Another problem we have that, community do not understand what the mental health problem and or disease. So, they go for traditional healers, which also play a big role in the community, and we are trying to assess …the rehabilitation centre. If we can also train them* (staff and traditional healers)*, will be fantastic, so that they can refer the mental* patients.” (KII-1-4:29-5:11min)

People often equate mental health problems with madness. Only severe mental health problems with serious behavioural abnormalities are considered as such and are called *wali*. *Wali*s are misunderstood in society and are not generally given care or medicines. Children throw stones at them and yell “*wad waalan tahay*” means “You are mad; you!”.

The poor perception of mental health problems prevails even among healthcare providers. Case vignettes of schizophrenia, depression, anxiety and ADHD (translated into Somali) were read to the health workers. They did not consider ADHD, depression, or anxiety as diseases, nor did they consider any treatment necessary for these problems. No psychotropic drugs were available in most of the PHC centres. Some centres did not collect information on mental health problems for the health information system, while in others, the appropriate row in the health information form was labelled “zero” meaning no patients with mental health problems were reported at those facilities. This suggests that the attending physicians did not have the ability to diagnose mental health problems.

People in general, including 75–100% of our participants, believed in black magic, *jinn* and evil spirits, which can cause mental health problems. This includes even support staff of an international organization working in Somalia in the health sector. Their logic is that when an otherwise physically fit person develops mental health problem, that must be *jinn* as the person did not have any problem (physical) before.

#### Mental health care

Mental health services are largely absent in the health care delivery system of the country except in few places mostly in the north. There is a huge shortage of qualified mental health personnel in the country. This may result from a lack of emphasis on mental health problems in the medical curriculum, lack of training opportunities in mental health problems and, more importantly, the apparent belief among the community as well as policymakers and health care professionals that there is not much need for mental health care. The private clinics mostly did not have any mental health care facility nor were they interested in providing such care to health-care seekers. When patients with mental health problems came to public hospitals, the attending healthcare provider usually told them, “We have nothing for you. Look for another place.” There are around six psychiatrists and 25 psychologists throughout Somalia. Even many regional public hospitals do not have a psychiatrist because they cannot afford to hire one. There are no beds or wards for treating patients with acute mental health problems either, as explained by the director of a regional hospital.

*“We don’t have psychiatrist here … , Last year we have psychiatrist, IOM (International Organization of Migration) was supporting us. After that the project was finished and he left. …[Interviewer: How do you manage your patient now?] We have doctor, who is a neurosurgeon... They are so aggressive, they are fighting, so we cannot keep in the hospital. … [Interviewer: you send them?] We refer to that facility*” (ID-2-0:14-2:29mins).

Later we visited the facility and found no psychiatrist and no regular staff for mental health care. It was just an isolation centre for those with mental health problems, who cannot be managed at home, where the rights of the patients were largely ignored.

The country does not have any mental health act. Under a new initiative, the Government was developing a new mental health policy and the strategy for 2019–2022 needed review and updating. While armed conflict continued, the Somali population also faced climatic shocks such as drought and flash floods. However, mental health problems are yet to get attention even from the humanitarian organizations in Somalia. One of the key informants echoed this.

*“We have lot of problem with increase in emergencies in the country. We do not have lot of focused mental health activities within the humanitarian settings. Now we have all these responses to emergency drought but with shelter and food, but no one is looking to mental health and psychosocial support aspect to the humanitarian response, which is also increasing creating lot of problem for us.*” (KII-1-6:50-7:20min)

*“The health worker even, instead of the community, the health workers even don’t know how to deal with mental, what mental illness. We have insufficient data. We don’t have unified HMIS system for mental health and also now we are facing lot of problem with the difficulty of referral pathway for clients or patients with MNS conditions.*” (KII-1- 7:20-8:40 mins)

Technically, many doctors who were aware of mental health problems did not even assess the mental status of the patients. Treatments were given based on clinical diagnosis, but no patient was seen to have been diagnosed with a mental health problem. The doctors were seen to change the diagnosis to a mental health problem when medicines prescribed for the initial diagnosis failed to provide any improvement. The views of the director of a regional hospital substantiate this observation.

“*We are all doctors. We don’t focus (on) the patient for mental (health problem). We just focus with the patient; what happened, are you seek, there are anything you eat, we do thing (diagnose this way). After we finish 7 days or 6 days, he will come back and say I will not take this medicine, I don’t need it. That’s the way we understand he is mental* problem. (ID-2-23:30-23:53min)

We observed neurosurgeons working as neurologists and psychiatrists and treating patients with mental health problems. One such neurosurgeon confirmed that, “yes, most of my clients, yeah, most of my patients are with mental health (problems)”.

Only *wali*s were considered as having mental health problems. When a *wal*i or drug user cannot be managed at home and cannot be helped by an *Aas-shefa* (traditional healer), the person would be taken to a regional hospital, doctor (usually a neurologist or neurosurgeon) or a rehabilitation centre for treatment. The rehabilitation centres also often do not have any psychiatrists. They restrain (sometimes with chains) and isolate patients to protect their family and friends and prevent the patient from taking drugs, committing suicide or self-harming. A focal point for mental health of a state Ministry of Health explained.

*“There is a lot of places, whether it is private, or partially public, people are being, they are looked after, you know feed them, they have somewhere to sleep but no treatment*.” (KII-2-2:18-2:33 min)

**Fig. 1:**
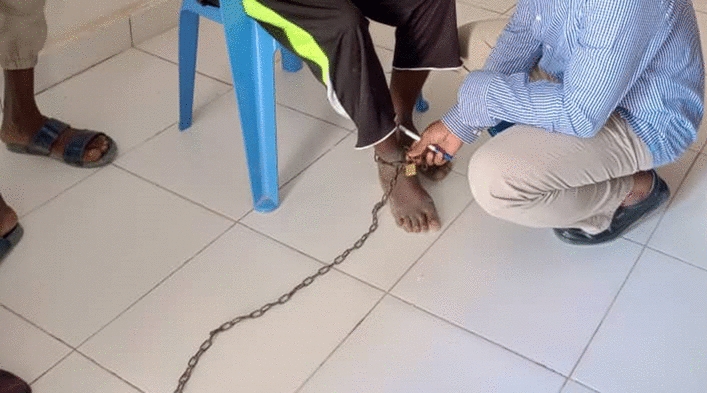
Patient chained at the ankle in a health centre

During our study, one patient was seen chained at a health centre (Fig. 1). The family members informed that the key to the lock was lost and as such the patient was not freed.

However, some facilities were providing good-quality mental health care. We had a discussion with two young diaspora psychologists who were operating such a private counselling centre. The number of such facilities, however, is not enough to meet the need and some of these facilities were struggling to keep open as the demand for good-quality mental health care among those seeking such care was low. Some diaspora doctors reportedly helped provide mental health care through telepsychiatry and some regularly visited some centres every 3–4 months to provide mental health care.

##### Care-seeking behaviour

Understanding care-seeking behaviour is important to develop a service delivery model. We found that most people, even educated ones, visit traditional healers (called sheikh) or *Aas-shefa* when they get sick. This is because they trust *ulema* (religious scholars). These traditional healers also charge less and use prayer and recitation from the Koran, which are culturally preferred by many Somali people. This is also true for patients with mental health problems.

*“They have physical complaints or acted abnormally to themselves, people take them to traditional healers (who often treat) by* (recitation of) *the Quran, sheikh and those, those people, they took them. We have traditional healers in Somalia, they are playing a big role. They have lots of mental patients. They call them, ‘no’, they have* jinn *or* sixir *(black magic). …They don’t refer, because they also don’t have training what is mental health disease*”. (KII-1-45:33-46:59)

Care for patients with mental health problems, as revealed during our discussion, also depended on the knowledge of mental health issues and the economic status of the family. Wealthy people usually kept the mental health status of their family members secret and looked for treatment elsewhere. These people go abroad to, for example, Ethiopia, Kenya or India to seek treatment for their family members. The poorer section of society tended not to seek care for their mentally ill family member, presumably due to a lack of knowledge or awareness. People were also observed to be reluctant to seek care for mental illness because of stigma. Sometimes, people try to buy over-the-counter medicines before seeking care from a service provider.

#### Cost of mental health care

Care-seeking behaviour was a complex issue with many factors playing a role. One important factor was cost, which affected the choice of care provider. Some people hold negative views about conventional medical practitioners as they are perceived as giving unnecessary tests and medicine for financial benefit.

“*You pay five dollars to Qur’anic healer; you get next seven days free. But if you go to the doctor, you pay the first 10 dollars for doctor’s card* (prescriptions), *then it will take more than 100 dollars in one hour. You pay in the laboratory, ultrasound. They will charge you even if you don’t need ultrasound, they put your money to ultrasound”.* (FGD-6-18:21-18:46min)

The local residential facilities which admit mostly substance use patients and people with severe mental health problems vary in their charges.

*“Most of facilities for mental health, (treat) by admission, monthly for around 100-150 (US) dollar per patient but that always can be changeable because some of them do not take that much*.” (KII-1-41:41-42:05min)

Psychologists in Somalia were understood to charge around US$ 25–40 per psychotherapy session. The cost of psychotherapy was a deterrent to uptake as it usually takes several sessions for a complete remedy. However, people in general are not familiar with this type of treatment.

*“Basically, they charge 25 per session. People don’t understand what psychotherapy and counselling is. The community even don’t understand. If you don’t give them medication, they don’t believe there is help with only talking*”. (KII-1-36:37-37:02min)

#### Referral to mental health care

Understanding how people access mental health care is important to develop an appropriate programme. There were no referral guidelines, nor was there any law for referral of patients with mental health problems. Furthermore, anyone can call themselves a doctor and treat patients. Even the psychologists prescribed medicines and used the title of “medical doctor” in front of their names. The lack of psychiatrists in Somalia also played a role in the poor referral practice for patients with mental health problems.

*“They* (general practitioners) *are not referring to the psychiatrist, they refer it to neurology. There is lack of psychiatrists at country level, so who is near to them is the neurologist, so they refer to neurology doctors. So, most neurology doctors in the country, they work as psychiatrist. You cannot say every doctor will do it*" (KII-1-42:39-43:37min)

A female psychiatrist in a private hospital did not see a single patient while she was there. This was partly because patients with a mental health problem that is not very severe often prefer to be labelled as *nerva* (a local term for symptoms of mostly anxiety or depression) and see a neurologist. Unless the neurologist refers them, they would prefer not to see a psychiatrist for treatment. Even when referred, these patients may not seek care from a psychiatrist because of the stigma surrounding mental illness. This phenomenon was observed even in the largest public hospital in the country in Mogadishu as narrated by a psychiatrist there.

“*That issue was even in our largest, our referral hospital. They started (a) psychiatry clinic, and no one usually go to psychiatry clinic, instead they go to neurologist. Even the psychiatrist went to neurologist, and they ask them why do you have these patients? Please refer, even you are not get paid. Your center is big, it is not private, you are not getting something, anything, it is, except workload. …Still they kept doing*”. (KII-3-48:52-49:26 min)

The same key informant said that in Somalia, neurologists were delivering services before psychiatrists. So, people think that they are the qualified ones for the treatment of mental health problems. He also recalled that he used to work with a neurologist, who only used to refer the difficult cases.

However, such referrals existed for care-seeking in some places such as between one *Aas-shefa* and another; a physician and an *Aas-shefa*; a private counselling service and a neurologist who manages patients with mental health problems; a neurologist and a counselling provider; a neurologist and a psychiatrist in complicated cases; and a physician and a psychiatrist or neurologist. Rarely, young patients contacted mental health care providers through the internet and received online services.

#### Stigma and discrimination

How the individual, family and community perceive a disease and whether it is stigmatized have important implications for programme development and use [27, 28]. The word “mental” was highly stigmatized in Somalia even among health care providers. People usually do not go to a psychiatrist because of stigma. One of the key informants, a psychiatrist, shared his experience.

“*Initially, he was talking about psychiatry, no one showed up (at) the (*psychiatry) *clinic. Then … they started saying that they do neuropsychiatry, … because our mhGAP treats people, you know. Whenever they started treating epilepsy… then patients who had somatic part of depression* (showed up) *…. Then the clinic become busier, because patients (with) somatic (complaints) who had either anxiety or depression showed up*.” (KII-3-47:45-48:38min).

Stigma was also found in doctors. Very few doctors were willing to have a career in psychiatry because of stigma. A focal point for mental health at a state ministry of health explained.

“*We have* (a) *lot of doctors. Even with the University they don’t have mental health doctors…. We keep advocating (to open a department for mental health) for them. But people (doctor), they don’t want it because there is lot of stigma. Even if you work with mental health people, they will stigmatize you. They will think, you know, you have an issue as well*.” (KII-2-14:17-14:40mins)

Stigma about mental health often affects every aspect of patients’ lives until they die. During one in-depth interview, a lady who had established a mental health centre in 2005, said that sometimes she had picked up patients from the road without knowing who they were. To illustrate how stigma can be detrimental to people’s lives, she recalled that during the COVID-19 pandemic, she had contacted the families of patients who died of COVID-19 in her health centre. The relatives of seven people came and buried the body. However, six people remained unidentified and were buried with the permission of the local government.

Stigma also appeared to be a barrier in the integration of mental health and psychosocial support within PHC services. A senior official working for a state ministry of health mentioned that when they were integrating mental health in PHC services, which were providing services for maternal and child health, the staff working in these facilities resisted saying-, “No, no, mad man can kill the (a) woman.”.

#### Tools

We did not come across any activity or tool to raise awareness, reduce stigma, promote mental health conditions as a disease, improve self-care and seeking appropriate care. We felt the need of such a tool that can be used by community people, community health workers, traditional healers, and community leaders including cultural or religious leaders and schoolteachers, which would help them to understand when anyone’s mental health condition can be considered as abnormal and as a disease and like other disease it can also be treated at PHC.

Pervasive unawareness, stigma, and neglect everywhere, from family to policymakers along with a lack of trained health personnel play a role in keeping mental health problems on the rise. A service delivery model addressing all the issues found involving all stakeholders is understood as an urgent need.

## Discussion

The Somali population, having been in a protracted humanitarian crisis for over 3 decades, have endured extreme suffering. This is reflected in the mental health status of the people in a small recent study. Our qualitative study is the first of its kind in Somalia aiming to understand the mental health problems of Somali people and the status of mental health services in the country. We believe that the findings will enhance our understanding of mental health in Somalia and provide information that may help design an appropriate health system response to mental health in the country. Given our findings, this response would need to address the harmful norms and practices in the community with regard to mental health; destigmatize mental health and support compassion and care for mentally ill people; counter misinformation on mental health in the community; and more importantly, change the perception of health service providers about mental health problems and mental health care in the country.

We found that nomadic inheritance, clan membership and kinship provide some informal support for mental health in the community but at the risk of political and armed conflicts amongst the warring groups often instigated by clan membership. Our findings concur with those of an ethnographic study among the Somali population in Ethiopia which showed how kinship helped support mental health and care for people with mental illness despite many challenges [29]. We also support their suggestion of using “kinship-based forms of social organization” in humanitarian aid and health care, including mental health care, as most people depend on informal support in times of need [30].

The prevailing insecurity in the country has resulted in huge outmigration, especially of skilled workers, from Somalia. We found that the Somali diaspora, most of whom live in Europe or the United States, were willing to help their people and are already working in many areas of the health sector where the security situation permits, such as in Somaliland [31]. Our findings support the importance of integrating interested members of the Somali diaspora in the design and delivery of future health programmes in the country, especially mental health care. The strong and emotional attachment that members of the Somali diaspora have for their country and people [29] supports the argument for involving this community in the health development programme in the country.

Interestingly, we found that most Somali people, including health personnel and policymakers, were not aware of the spectrum and nature of mental health problems and equate mental health problems with severe abnormal behaviour. This may explain why mental health is so stigmatized in the country. Our findings concur with those of a study among Somali refugees New Zealand in about 20 years ago [32]. This shows that the perception of Somali people on mental health problems has not changed over the decades. However, we found some changes among people who met mental health care providers from the Somali diaspora. This highlights that providing proper care, educating the Somali people in their language, and providing correct information can change perceptions. This again supports the inclusion of people among the Somali diaspora in programmes to increase awareness and improve mental health care in the country. The pervasive stigma affected various aspects of the life of people with mental health problems. They often faced discrimination and humiliation and were forced to lead an isolated life. This stigma deterred people from being diagnosed and seeking proper help. It also discouraged young physicians from pursuing a career in mental health or psychiatry. As a result, the country does not have enough trained mental health care professionals in the country. This scarcity hinders the integration of mental health care into the PHC programme of the country. Our findings are in line with a study in a Somali community in the United Kingdom where participants mentioned how mental problem was a taboo issue, that people were afraid of persons with mental problems and how such people were kept hidden from the public [33].

**Fig. 2:**
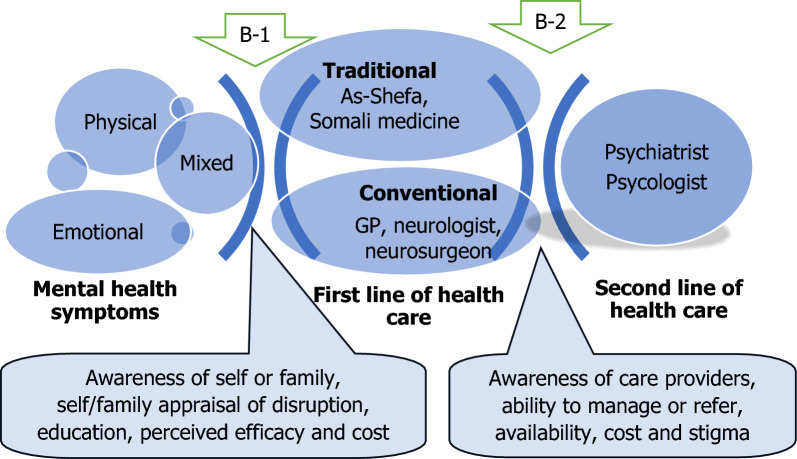
Pathway to mental health care: factors, actors and barriers. B-1 barrier at the individual/family level; B-2 barrier at the service provider level

Our findings showed that care-seeking for mental illness is limited and often delayed for years as mentioned by others [9]. Based on our findings, we conceptualized a care-seeking pathway and identified two levels of barrier to mental health care: B-1 at the individual and family level and B-2 at the service provider level (Fig. 2). Our framework is almost similar to ‘Goldberg—Huxley Model’ but with three levels of care and two levels of filters or barriers instead of their five and four, respectively [34]. This difference is due to the lack of availability of mental health care at all levels of care, which sometimes we see at only one level. The first level of barrier, B-1 is unawareness of the individual or family who often decides on their self-appraisal based on the disruption caused by the person with a mental health condition. This appraisal often influenced by their education. When it causes substantial disruption in others' lives, the perceived efficacy of a school of treatment comes into play. At level two (B-2), when they reach a particular category of care providers, their ability to diagnose and manage or refer along with stigma and cost determine whether the patient will receive care given by a psychiatrist or psychologist. This in turn also depends on the availability. These factors work in a complex way for the care seekers to reach an appropriate level of mental health care. Understanding these factors and their relationship with each other needs to be addressed when formulating a mental health care programme in the country. Raising awareness, improving the identification of people with mental health problems, and improving their care-seeking are important steps towards the effective provision of mental health care [35]. This can be facilitated by a tool to raise awareness that can be used by different actors such as family and community at level one (B-1).

As mentioned before, except in Somaliland, the security situation was unpredictable and challenging in all study areas in Somalia. Despite the huge security concern, we tried to ensure the trustworthiness of our findings. We used five different methods such as IDIs, FGDs, IDs, CSs and observations to collect data in six places in Somalia with different security situations and triangulate our data. This method and data triangulation helped us to glean credible information. We detailed our methodology and findings along with the challenges. The consistency across the methodological rigour and the findings contributed to the dependability of our research. Our long familiarity with qualitative research allowed us to understand the situation without being judgemental. In addition, the availability of records and field notes ensured the confirmability our findings. We believe our findings are transferable to other security-compromised similar sociocultural settings.

## Strengths and limitations

The prevailing security situation and movement restrictions were a challenge to carrying out this study. As mentioned before, we could not collect in-depth data from the host community and IDPs in their natural settings as talking to people using armed escorts could endanger the people we wanted to talk to afterwards. However, FGDs with respondents with a wide range of Somali sociodemographic backgrounds, our observation of the different study sites, a large number of informal discussions with different stakeholders and more importantly three case studies greatly compensated for the caveat.

The flexibility and emergent nature of qualitative data analysis allowed us to analyse data to have a credible understanding of ‘what is going on’, why and how [13].

In our study, we did some translation and back translation into English from Somali, this might have introduced some weaknesses in our understanding. However, one of the authors was a Somali national who was closely involved in this process and helped in minimizing biases. Using more than one method of data collection and triangulation also contributed to the accuracy of our data.

We observed a substantial dependency on donors across the health sector and some incentives was generally expected for participating. This might have introduced some biases. Despite these limitations, a strength of our study is that it is the first of its kind in Somalia carried out by researchers with long experience in qualitative research and included a wide range of methods and informants, from the policy level down to the service level.

## Conclusion

Mental health care is largely underfunded, undervalued and invisible in Somalia with inadequately trained health workers, supplies, budgetary support and infrastructure. There is an urgent need for a mental health programme, structure and system, and more trained mental health professionals. Given the unawareness and stigma about mental health, there is also a need for a tool to raise awareness about mental health problems and the importance of mental health care across the country among both the public and health workers.

Everyone has the right to health and appropriate care when needed. We believe that addressing the mental health problems in Somalia will also address social injustice and health inequality and thus some aspects of human rights.

## Data Availability

The data and recorded audios available from the corresponding author on reasonable request.
